# Taiwan’s Successful COVID-19 Mitigation and Containment Strategy: Achieving *Quasi Population Immunity*

**DOI:** 10.1017/dmp.2020.357

**Published:** 2020-09-11

**Authors:** Li-Chien Chien, Christian K. Beÿ, Kristi L. Koenig

**Affiliations:** Disaster Division and Emergency Medicine, Taipei City Hospital, Datong District, Taiwan; Institute of Hospital and Health Care Administration, National Yang-Ming University, Beitou District, Taipei City, Taiwan; University of California San Diego, La Jolla, CA; Emergency Medical Services, County of San Diego, Health & Human Services Agency, San Diego, CA; Department of Emergency Medicine and Public Health, University of California Irvine, Orange, CA

**Keywords:** COVID-19, emerging infectious disease, non-pharmaceutical interventions, SARS-CoV-2, Taiwan

## Abstract

The authors describe Taiwan’s successful strategy in achieving control of coronavirus disease (COVID-19) without economic shutdown, despite the prediction that millions of infections would be imported from travelers returning from Chinese New Year celebrations in Mainland China in early 2020. As of September 2, 2020, Taiwan reports 489 cases, 7 deaths, and no locally acquired COVID-19 cases for the last 135 days (greater than 4 months) in its population of over 23.8 million people. Taiwan created *quasi population immunity* through the application of established public health principles. These non-pharmaceutical interventions, including public masking and social distancing, coupled with early and aggressive identification, isolation, and contact tracing to inhibit local transmission, represent a model for optimal public health management of COVID-19 and future emerging infectious diseases.

Severe acute respiratory syndrome coronavirus 2 (SARS-CoV-2) was first reported to the World Health Organization in late December 2019.^1^ By the end of January 2020, early reports that the novel virus was not spread from person-to-person were disproven and nosocomial infections were occurring in hospitals in Wuhan, China. Given the absence of a vaccine or proven treatments for its associated disease, coronavirus disease (COVID-19), the entire global population is susceptible, and, without public health intervention, substantial viral transmission and mortality are certain.

Public health measures taken to curb the spread of the virus have varied worldwide. While some countries, such as Sweden, have apparently embraced a “herd immunity” model, this strategy typically requires infection of approximately 70% to 90% of a given population, which would result in significant morbidity and mortality. Other countries have engaged in variable degrees of non-pharmaceutical interventions (NPIs), such as public masking and physical distancing. Some regions have also implemented strict quarantines, for example, in Wuhan, China. NPIs are proven public health measures to contain viral spread; however, these types of social distancing and other protective strategies are ineffective unless implemented early and comprehensively. When cultural, religious, or family exceptions are permitted, social distancing strategies are severely weakened. This occurred in the past, such as during the 1918 influenza pandemic.^2^

Social distancing strategies require a change in human behavior, which is challenging in many cultures. Additionally, lockdowns, while effective in inhibiting viral transmission, are catastrophic for the economy, and can contribute to negative health consequences. Their implementation is also associated with deleterious mental and physical health outcomes.

## REPORT

### Herd Immunity

Herd immunity is a form of indirect population protection from infectious disease that occurs when a sufficient percentage of people become immune, either through effective vaccination or via previous infection. Theoretically, to achieve herd immunity in Taiwan’s population of 23.8 million, at least 70% of the population (16.7 million) would have to be infected within 8 weeks. This would likely result in significant morbidity and mortality, and is not currently achievable.

During a pandemic, populations can be triaged into 5 distinct categories using the SEIRV model: (1) Susceptible (not exposed, but susceptible); (2) Exposed (infected, but incubating the disease and not symptomatic or contagious); (3) Infectious (contagious); (4) Removed (non-contagious and immune by recovery or non-contagious by death); and (5) Vaccinated or on prophylactic medications (protected).^3^ In the early stages, there will be insufficient numbers of persons in categories 4 or 5. Thus, the vast majority of the population is susceptible to the virus, herd immunity is not achieved, and the outbreak cannot be contained.

**FIGURE 1:**
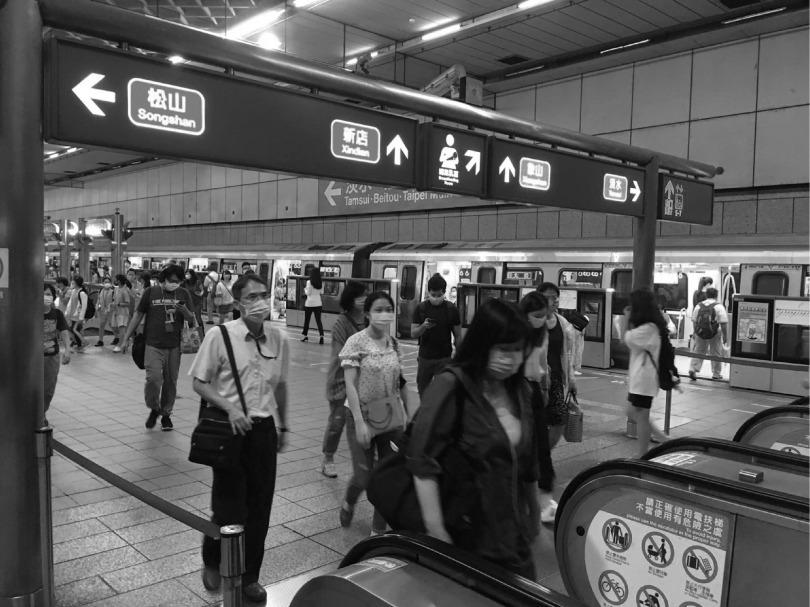
Public Masking Compliance in the Taipei Mass Rapid Transit (MRT) Metro System.

As herd immunity will likely be unachievable prior to vaccine development and subsequent global vaccination, we describe and analyze an alternative strategy implemented in Taiwan that has thus far proven to successfully halt viral transmission, without economic shutdown (Figure 1). Despite no widespread home quarantines, as of September 2, 2020, Taiwan has reported only 489 cases and 7 deaths in a population of over 23.6 million,^4^ with no documented cases of local community spread for the last 135 days – nearly 10 incubation periods.

### Achieving Quasi Population Immunity

The term *quasi-* is defined as “seemingly” or “apparently but not really.”^5^ Hence, it is a useful term to describe temporary immunity – resistance to disease either naturally or through acquired means – at the population level. This strategy is effective at bridging the gap until proven therapeutics and safe and effective vaccination can be implemented. How then did Taiwan achieve *quasi population immunity* without ever initiating a widespread economic shutdown? A mere 130 kilometers from Mainland China by sea and air, Taiwan braced for the influx of approximately 500 000 travelers anticipated to return from Chinese New Year celebrations on January 25, 2020, shortly after the Chinese Government locked down Wuhan on January 23. Experts initially predicted this migration would result in millions of infections. However, subsequent to the 2003 SARS epidemic, Taiwan had developed an extensive infrastructure to combat emerging infectious diseases. An outbreak of influenza in February 2016, resulting in approximately 2000 critical cases, led to further enhancements in the country’s pandemic management system.^6^ Thus, contrary to forecasting, the number of cases in Taiwan rose only slowly. After reaching 166 cases by February 17, the number plateaued.^7^

Taiwan’s strategy to contain COVID-19 included implementation of NPIs to flatten the epidemic curve, while concurrently improving rapid testing capacity. Hospitals across the country implemented strict visitor restrictions and erected outdoor triage tents. Importantly, the general populace donned government-provided simple surgical masks when leaving their homes to help inhibit asymptomatic spread (see Figure 1). Rigorous contact tracing using mobile phones to confirm compliance with quarantine and isolation was effective and revealed that household members and intimate contacts were responsible for most viral transmissions. Community transmission was not a major factor.

Initially, the Taiwanese Government advised citizens to avoid wearing masks if healthy. However, public masking became essential after asymptomatic transmission was reported, prompting the government to issue a mandate to wear masks in public places on April 4, 2020. To augment supplies of personal protective equipment, the government rapidly organized and funded 60 lines of surgical mask production, resulting in the daily production of 10 million masks by April 2020. The Taiwanese Government now provides the general public with 3 surgical masks per week. In addition, Taiwan has provided more than 16 million medical masks to support medical professionals around the world under the umbrella of the “Taiwan Can Help” initiative.^8^

## DISCUSSION

We hypothesize that containment strategies, codified by the Identify-Isolate-Inform (3I) model,^9^ and mitigation efforts, implemented using NPIs (public mask-wearing, physical distancing, and hand and surface hygiene), created *quasi population immunity* in Taiwan. The level of population immunity required to effectively contain spread of the disease is a function of R_**0**_, the basic reproduction number. By lowering the effective reproductive rate with NPIs, the population immunity threshold is much lower. Specifically, it is likely that universal public masking, combined with open outdoor environments where physical separation is respected, successfully inhibited infection transmission despite no “lockdown” or closure of schools, transportation, or restaurants. Taiwan even continued baseball games with modifications to ensure physical distancing and mask-wearing by spectators. As of September 2, 2020, the outbreak remains under control.

### The Future of Population Immunity

COVID-19 pandemic mitigation and containment strategies will inform management of future public health emergencies resulting from emerging infectious diseases, prior to the development of vaccinations or natural herd immunity. Transmittable illnesses, such as measles, mumps, and varicella, have been controlled through vaccine-based herd immunity. Before the creation of these vaccines, natural herd immunity was effective in protecting a subset of the population while leaving others vulnerable. However, the destabilizing effects of COVID-19 on health care systems and the number of infected individuals required to be infected make achieving natural herd immunity unfeasible. With growing pressure to reopen economies prior to vaccine development, strategies to create *quasi population immunity* can be implemented to control a pandemic.

### Limitations

Mortality data were obtained from the Taiwan Centers for Disease Control. As with many other international databases, out-of-hospital deaths were not always included; therefore, the death numbers are likely underestimated.

## CONCLUSION

In order to successfully re-open economies without accelerating local outbreaks, public health officials must ensure prompt containment using the 3I (Identify-Isolate-Inform) Tool. This encompasses rigorous identification, immediate isolation of individuals, and informing public health to implement contact tracing. Asymptomatic exposed individuals must be quarantined and ill individuals promptly isolated. Identification of susceptible exposed persons must be coupled with NPIs that include public masking and physical distancing, with a special emphasis on avoiding cohorting of individuals in enclosed spaces.

Taiwan’s success in achieving outbreak containment through the implementation of early and aggressive containment and mitigation strategies, despite close proximity to ground zero of the novel virus, can be conceptualized as the initiation of *quasi population immunity*. Essentially, Taiwan has rendered the population immunity percentage component less relevant by implementation of a specific set of NPIs, thereby achieving control of viral spread. The Taiwanese model is useful to inform public health experts and policy-makers worldwide as we work together to reduce morbidity and mortality from this rapidly evolving pandemic.

## References

[1] World Health Organization. Rolling updates on coronavirus disease (COVID-19). 2020. https://www.who.int/emergencies/diseases/novel-coronavirus-2019/events-as-they-happen. Accessed May 16, 2020.

[2] Hatchett RJ , Mecher CE , Lipsitch M. Public health interventions and epidemic intensity during the 1918 influenza pandemic. PNAS. 2007;104(18):7582-7587. 17416679 10.1073/pnas.0610941104 PMC1849867

[3] Burkle FM Jr . Population-based triage management in response to surge-capacity requirements during a large-scale bioevent disaster. Acad Emerg Med. 2006;13(11):1118-1129. 17015415 10.1197/j.aem.2006.06.040

[4] Taiwan Centers for Disease Control. Taiwan National Infectious Disease Statistics System. 2020. https://www.cdc.gov.tw/En/Bulletin/List/7tUXjTBf6paRvrhEl-mrPg. Accessed May 16, 2020.

[5] “Quasi-.” New Oxford American Dictionary. 3rd ed Oxford, England: Oxford University Press; 2010.

[6] Chien LC , Yeh WB , Chang HT. Lessons from Taiwan. CMAJ. 2003;169(4):277. PMC180637 12925410

[7] Taiwan Centers for Disease Control. Taiwan National Infectious Disease Statistics System. 2020. https://www.cdc.gov.tw/En. Accessed May 16, 2020.

[8] Taiwan Can Help. WHO can help? 2020. https://taiwancanhelp.us/. Accessed May 17, 2020.

[9] Koenig KL , Beÿ CK , McDonald EC. 2019-nCoV: The Identify-Isolate-Inform (3I) Tool applied to a novel emerging coronavirus. West J Emerg Med. 2020;21(2). 10.5811/westjem.2020.1.46760 PMC7081861 32191174

